# Thermal proteome profiling and proteome analysis using high‐definition mass spectrometry demonstrate modulation of cholesterol biosynthesis by next‐generation galeterone analog VNPP433‐3β in castration‐resistant prostate cancer

**DOI:** 10.1002/1878-0261.70009

**Published:** 2025-02-26

**Authors:** Retheesh S. Thankan, Elizabeth Thomas, Mehari M. Weldemariam, Puranik Purushottamachar, Weiliang Huang, Maureen A. Kane, Yuji Zhang, Nicholas Ambulos, Bi‐Dar Wang, David Weber, Vincent C. O. Njar

**Affiliations:** ^1^ Department of Pharmacology University of Maryland School of Medicine Baltimore MD USA; ^2^ Isoprene Pharmaceuticals, Inc. University of Maryland Baltimore BioPark Baltimore MD USA; ^3^ The Center for Biomolecular Therapeutics University of Maryland School of Medicine Baltimore MD USA; ^4^ Department of Pharmaceutical Sciences University of Maryland School of Pharmacy Baltimore MD USA; ^5^ Division of Biostatistics and Bioinformatics University of Maryland Marlene and Stewart Greenebaum Comprehensive Cancer Center Baltimore MD USA; ^6^ Department of Microbiology and Immunology University of Maryland Marlene and Stewart Greenebaum Comprehensive Cancer Center Baltimore MD USA; ^7^ Department of Pharmaceutical Sciences, School of Pharmacy and Health Professions University of Maryland Eastern Shore Princess Anne MD USA; ^8^ Marlene and Stewart Greenebaum Comprehensive Cancer Center University of Maryland School of Medicine Baltimore MD USA; ^9^ Department of Biochemistry and Molecular Biology University of Maryland School of Medicine Baltimore MD USA; ^10^ Present address: School of Chemistry and Molecular Biosciences The University of Queensland St. Lucia Australia

**Keywords:** cholesterol, CYP51A1, lanosterol synthase, next‐generation galeterone analog, prostate cancer, thermal proteome profiling

## Abstract

Cholesterol (CHOL) homeostasis is significantly modulated in prostate cancer (PCa) suggesting an active role in PCa development and progression. Several studies indicate a strong correlation between elevated CHOL levels and increased PCa risk and severity. Inhibition of CHOL biosynthesis at different steps, including lanosterol synthase (LSS), has shown significant efficacy against both hormone‐dependent and castration‐resistant PCa. Earlier, we reported proteasomal degradation of androgen receptor (AR)/AR‐Vs and Mnk1/2 as the primary mechanisms of action of VNPP433‐3β in inhibiting PCa cell proliferation and tumor growth. Through thermal proteome profiling, comparative proteomics and cellular thermal shift assay, we identified VNPP433‐3β's ancillary effect of lowering CHOL by binding to LSS and lanosterol 14‐alpha demethylase, potentially inhibiting CHOL biosynthesis in PCa cells and tumors. Additionally, in conjunction with our previously reported transcriptome analysis, proteomics reveals that VNPP433‐3β modulated upstream regulators and pathways critical for PCa stem cell maintenance and recurrence. The inhibition of CHOL biosynthesis by VNPP433‐3β reinforces its multifaceted effects in PCa across all stages, highlighting its potential as a single‐agent therapy. Achieving reduced CHOL levels aligns with better treatment outcomes, further substantiating VNPP433‐3β's therapeutic potential.

## Graphical abstract

Elevated level of cholesterol is positively correlated to prostate cancer development and disease severity. Cholesterol‐lowering drugs, such as statins, are demonstrated to inhibit prostate cancer. VNPP433‐3β interrupts multiple signaling and metabolic pathways, including cholesterol biosynthesis, AR‐mediated transcription of several oncogenes, mRNA 5′ cap‐mediated oncogenic protein synthesis (by interrupting eIF4E‐Mnk1/2 signaling) and induces apoptosis in prostate cancer cells.

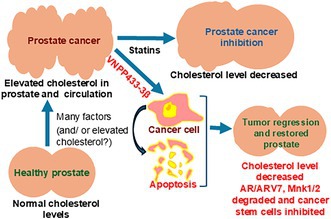

**Abbreviations**
22Rv1: CWR22Rv1
433‐3β: VNPP433‐3β
AR: androgen receptor
CETSA: cellular thermal shift assay
CHOL: cholesterol
CRPC: castration‐resistant prostate cancer
DPN: diarylpropionitrile
DTX: docetaxel
eIF4E: eukaryotic translation initiation factor 4E
ENZ: enzalutamide
FBS: fetal bovine serum
GAL: galeterone
HD‐MS: high‐definition mass spectrometry
HMGCR: HMG CoA reductase (3‐hydroxy‐3‐methyl‐glutaryl‐coenzyme A reductase)
LDM: lanosterol 14‐alpha demethylase (CYP51A1)
LSS: lanosterol synthase
Mnk1/2: MAP kinase‐interacting serine/threonine‐protein kinase 1 and 2
MTT: 3‐(4,5‐dimethylthiazol‐2‐yl)‐2,5‐diphenyltetrazolium bromide
NGGA: next‐generation galeterone analog
OSC: oxidosqualene cyclase
PCa: prostate cancer
PMSF: phenylmethylsulfonyl fluoride
PSA: prostate‐specific antigen
RO: RO48‐8071
SREBP‐2: sterol regulatory element‐binding protein 2
TPP: thermal proteome profiling

## 1 Introduction

The role of cholesterol (CHOL), a precursor for androgen biosynthesis in prostate cancer (PCa), remains largely speculative though it was first reported more than eight decades ago [1]. A previous study on a cohort of 1997 healthy Norwegian men suggested a negative correlation of CHOL levels to PCa but cautioned the need for further studies [2]. However, several lines of evidence and numerous investigations suggest a strong association of higher CHOL levels with increased risk of PCa and disease severity [3, 4, 5]. Some argue that elevated CHOL levels could be a cause, while others contend it is a consequence of PCa. However, whether it is considered a cause or an effect, increased levels of CHOL are strongly associated with PCa progression and disease severity [6, 7] and decreasing CHOL levels using ezetimibe (inhibitor of CHOL uptake) significantly reduced androgen levels in prostate and retarded tumor growth in PTEN‐null mice [7, 8, 9]. Interestingly, transcriptome analysis shows upregulation of CHOL biosynthesis pathway in enzalutamide‐resistant LNCaP‐derived MR49F cells compared with parental LNCaP cells and blocking CHOL biosynthesis using simvastatin effectively countered enzalutamide resistance in castration‐resistant prostate cancer (CRPC) [10]. Inhibiting CHOL biosynthesis using lanosterol synthase (LSS) inhibitor RO48‐8071 (RO) significantly inhibited hormone‐dependent and castration‐resistant PCa cells *in vitro* and PCa tumor growth in *in vivo* tumor xenograft mice models [11] and breast cancer [12]. Additionally, several reports demonstrate that biochemical inhibition of LSS can significantly inhibit various cancers in preclinical models, including PCa [11, 13, 14, 15]. Increased accumulation of CHOL and fatty acids in peripheral blood is reported in a Chinese cohort of PCa patients and subsequent xenograft studies demonstrate a strong association of increased CHOL levels with increased risk of PCa progression from prostatic intraneoplasia to invasive PCa with an increase in PCa stem cells [16]. High‐fat diet is an established risk factor of PCa [17]. Furthermore, lowering serum CHOL levels through dietary interventions notably decreased both serum PSA levels and the estimated risk of PCa in men [9]. Therefore, mounting evidence suggests a potential positive correlation between CHOL levels and PCa incidence and disease severity.

Even with the availability of advanced medical care for patients, the absence of an effective drug to combat metastatic CRPC remains a significant challenge. As a result, PCa continues to rank as the second most common cause of cancer‐related death among American men and the second most prevalent globally in 2020 [18]. As of 2023, like previous years, PCa continues to stand as the most prevalent cancer type among American men, representing an astonishing 29% of all cancer diagnoses [19]. These statistics indicate a significant gap in translational medicine and deserve urgency in developing novel targeted therapies. As androgen receptor (AR) is a key transcriptional factor that governs the expression of several cell cycle proteins, hyperactivation and aberrant AR signaling is linked to most cases of PCa disease severity and increased mortality [20, 21, 22]. The unusual activation of AR signaling and its resurgence even after castration is a major hallmark of PCa in most clinical cases and hurdle to overcome it but could be a steppingstone toward taming the challenge with the advent of novel AR‐degraders [23, 24, 25].

Previously, our laboratory has developed galeterone (GAL), that advanced to phase III clinical trials in men with castration‐resistant PCa [26, 27]. Subsequently, we developed more potent next‐generation galeterone analogs (NGGAs), VNPP433‐3β (433‐3β) being a lead molecule [28, 29, 30]. Statins that inhibit HMGCR enzyme in the mevalonate (MVA) pathway and thus interfere with CHOL biosynthesis activate feedback loop involving SREBP2 and lessen the efficacy of statins in PCa [31] but a combination of statin (Fluvastatin) and GAL is reported to inhibit CRPC *in vivo* by preventing the activation of SREBP2 and the consequential restorative feedback loop [32]. We have determined that the primary mode of action of 433‐3β is to enhance the proteasomal degradation of AR (a ligand/androgen‐activated transcription factor) in PCa cells thereby interrupting AR signaling [25, 33]. Mechanistically, 433‐3β binds AR and facilitates MDM2‐mediated ubiquitination and subsequent proteasomal degradation of AR, thus preventing nuclear translocation of AR and AR‐mediated oncogenic transcription [25, 33, 34]. Additionally, it significantly decreased the protein levels of Mnk1/2 (MKNK1/2, MAPK interacting serine/threonine kinase 1/2), the only kinases known to phosphorylate eIF4E necessary for mRNA 5′‐cap‐dependent translation, thus regulating extensive oncogenic protein synthesis [25, 33, 35]. Our present understanding strongly suggests that 433‐3β inhibits AR‐mediated oncogenic transcription and Mnk1/2‐eIF4E‐mediated mRNA 5′ cap‐dependent protein synthesis in PCa [25].

To further extend our studies on its effects in other metabolic and signaling pathways of PCa, we performed thermal proteome profiling (TPP) and comparative proteomic studies in PCa cells treated with 433‐3β. Taking lead from TPP data, here, we report binding of 433‐3β to lanosterol synthase (LSS) and Lanosterol 14‐alpha demethylase (LDM, CYP51A1) and potentially inhibiting them, two key rate‐limiting enzymes in CHOL biosynthesis pathway [36]. We demonstrate that treating PCa cells with 433‐3β leads to decrease in CHOL levels in PCa cells *in vitro* and in xenograft tumor tissues of treated mice by potentially inhibiting lanosterol synthase and CYP51A1.

## 2 Materials and methods

### 2.1 Cell culture, chemicals and reagents

The human prostate carcinoma epithelial cell line CWR22Rv1 (22Rv1, Cellosaurus accession – CVCL_1045) that was certified mycoplasma‐free was obtained from ATCC (Manassas, VA, USA) and was used within 3 years of procurement. All cells used for the experiments were confirmed mycoplasma‐free prior to use. The cells were cultured in RPMI‐1640 medium containing 10% FBS (GIBCO) and 1% Pen‐Strep (10 000 U·mL^−1^; Life Technologies, Frederick, MD, USA) under standard conditions of 37 °C with 5% CO_2_ and grown to approximately 80% confluency. The culture medium was replaced every 48–72 h. The in‐house synthesis of GAL and 433‐3β were performed as reported [37]. DMSO (Sigma‐Aldrich, St Louis, MO, USA), RO (RO48‐8071; EMD Millipore Corp, Burlington, MA, USA), diarylpropionitrile (DPN), CHOL (Millipore‐Sigma, Burlington, MA, USA), enzalutamide (ENZ, CAS 915087331), docetaxel (DTX, Cat #45008; AstaTech, Bristol, PA, USA), and fine chemicals were purchased from Sigma, (St. Louis, MO, USA), unless otherwise mentioned. Anti‐LSS was purchased from Proteintech (Rosemont, IL, USA).

### 2.2 Cell viability assay

The 22Rv1 cells were seeded in each well (2500 cells/well) of a 96‐well plate and treated as indicated on second and fifth day with six replicates for each treatment. On the eighth day, the experiment was terminated, media removed, and MTT dye was added. Subsequently, the cells were incubated at 37 °C and 5% CO_2_ for 2 h, dye replaced with 100 μL DMSO, and readings were taken in microplate reader (Biotech ELX800, Thermo Scientific, Swedesboro, NJ, USA) at 570 nm. graphpad prism 9.0 software (GraphPad Software, Inc., La Jolla, CA, USA) was used to calculate Growth inhibitory concentration (GI_50_) based on a non‐linear regression curve fit.

### 2.3 Cholesterol assay in cells and tumor tissue

Fluorometric quantification of total CHOL in cells and tissue was performed using Amplex™ Red CHOL Assay Kit following manufacturer's instructions (A12216; Invitrogen, Waltham, MA, USA). The fluorescence was read in 96‐well plate in a Tecan Spark multimode microplate reader (Tecan Group Ltd., Mannedorf, Switzerland).

### 2.4 Cholesterol rescue assay

CHOL rescue assay was performed as reported previously [14] by estimating the cell viability after indicated treatment using MTT cell viability assay. CHOL, GAL, and 433‐3β dissolved in DMSO was added to 22Rv1 cells grown in 96‐well plates at indicated concentrations before estimating the fraction of viable cells.

### 2.5 *In vivo* study

The 22Rv1 xenograft tumor tissues from a previous study were used for estimating CHOL in PCa excised tumor [38]. Immunodeficient male NRG mice (NOD‐Rag1null IL2rgnull; 5–6 weeks old) were obtained from the Veterinary Resources, University of Maryland School of Medicine (Baltimore, MD, USA). The mice were housed in ventilated cages inside pathogen‐free animal rooms at 22 °C and 60% humidity and provided with 12 h of light and 12 h of dark cycle. The animals were provided with unlimited access to sterile pellets and water. After acclimatization for a week, tumors were induced by injecting 22Rv1 cells onto the left flank. All animal experiments were approved by the Institutional Animal Care and Use Committee (IACUC), University of Maryland School of Medicine, Baltimore (IACUC No. #0421007 dated 01/31/2022). The mice were treated with previously reported optimum preclinical doses of ENZ [39] and DTX [40]. Animal experiments were carried out per the humane use of experimental animals and as approved by the Institutional Animal Care and Use Committee (IACUC).

### 2.6 Molecular docking

The ligand 433‐3β was docked *in silico* to the crystalline structure of human lanosterol synthase (LSS), an oxidosqualene cyclase (OSC) and lanosterol 14‐alpha demethylase (CYP51A1) available in the protein databank PDB (PDB id 1W6K [41] and 6UEZ [42], respectively), using the algorithm autodock vina 1.1.2 [43]. First, the ligand used for co‐crystallization of the proteins and associated water molecules were removed. Next, the algorithm was employed to add polar hydrogens and Kollman charges to the protein. The grid box centered at 2.135, 27.699, and 3.831 for *x*, *y* and *z*, respectively, with grid points of 40 each in *x*, *y*, and *z* dimensions and default spacing as described previously [44]. Assuming a rigid structure of the protein, rigid docking was carried out using genetic algorithm with exhaustiveness of conformational sampling of 8 and all other parameters with default values. The molecular conformation with the lowest Gibb's free energy of binding (Δ*G*°) was simulated in BIOVIA Discovery Studio Visualizer, Dassault Systems, San Diego, CA, USA.

### 2.7 Immunoblotting and cellular thermal shift assay

The cell lysates were prepared by freeze–thaw in liquid nitrogen and heat block set at 25 °C from the treated and control 22Rv1 cells in PBS buffer containing 1× protease inhibitors (Roche, Indianapolis, IN, USA), phosphatase inhibitors (Thermo Scientific, Waltham, MA, USA), 1 mmol·L^−1^ EDTA and 1 mmol·L^−1^ PMSF (Sigma), and added 2× SDS/PAGE loading buffer (Thermo Fisher) [45, 46]. Protein (20–50 μg) was separated in 4–15% Mini‐PROTEAN TGX gels by electrophoresis in Mini‐PROTEAN system (Bio‐Rad, Hercules, CA, USA). The separated proteins were electroblotted onto PVDF membrane (Bio‐Rad), probed with Anti‐LSS antibody (13715‐1‐AP; Proteintech), and documented in iBright 1500 (Thermo Fisher Scientific). Cellular thermal shift assay (CETSA) was performed as previously reported [47]. Briefly, 22Rv1 cells were treated with 433‐3β (10 μm) or DMSO control for 4 h. The cells were washed and resuspended in PBS containing protease inhibitors. The cells were aliquoted in batches of 100 μL (1 × 10^6^ cells for each temperature), subjected to varying temperatures as designated in a thermal cycler (Applied Biosystems, Waltham, MA, USA) for 3 min followed by lysing by repeated freeze–thaw in liquid nitrogen, and heat block set at 25 °C with intermittent vortex. The cell lysates were centrifuged at 20 000 ***g*** for 20 min at 4 °C, discarded the pellet that contains cell debris, and protein aggregates. The supernatant fraction with soluble proteins was subjected to SDS/PAGE as mentioned and immunoblotted using anti‐LSS or anti‐CYP51A1. The bands were quantified by densitometry using image lab (Bio‐Rad), and graph was generated as a function of temperature vs relative band intensity to obtain the apparent melting curve in graphpad prism 9.0.

### 2.8 Thermal proteome profiling and comparative proteomics by high‐definition mass spectrometry

**Fig. 1:**
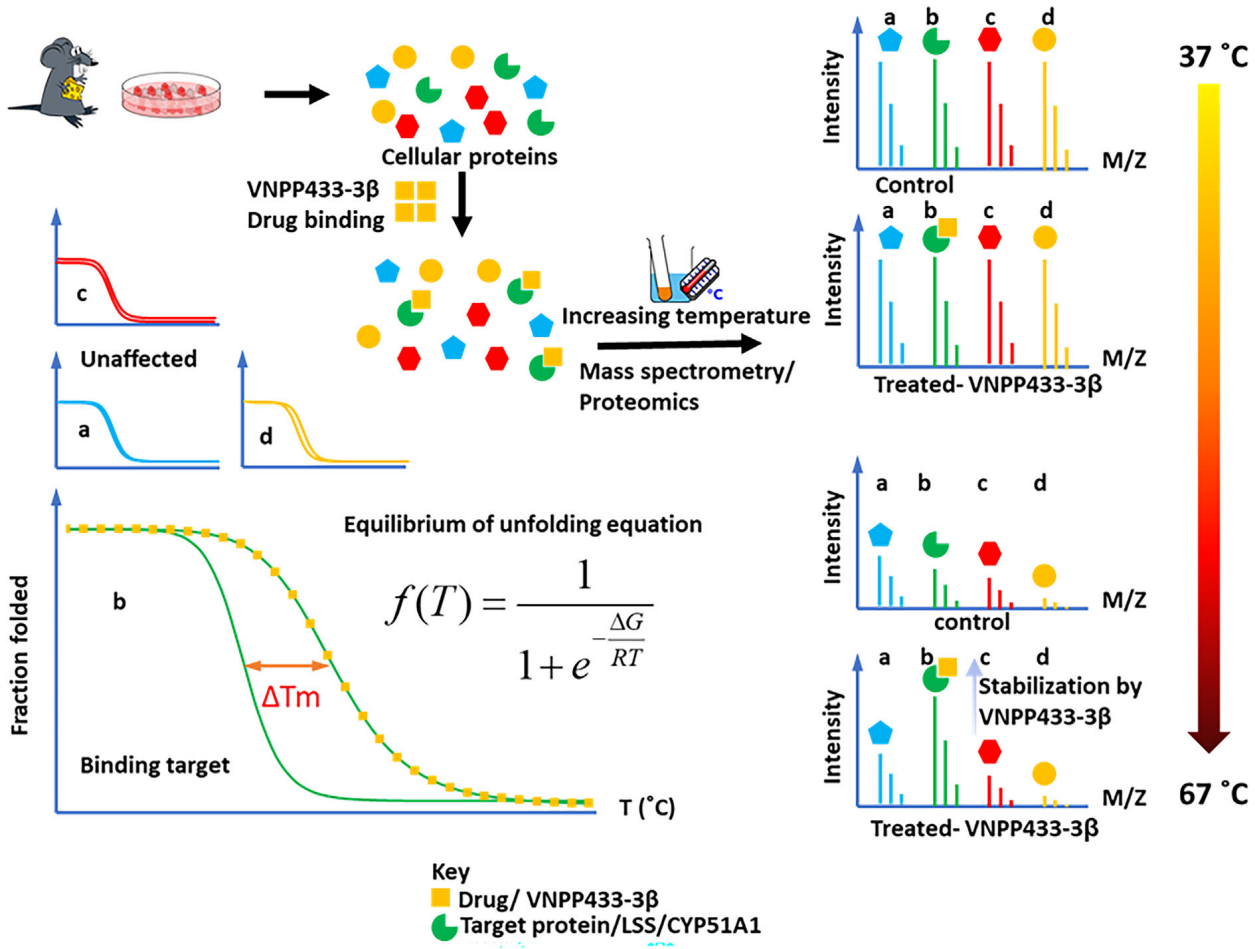
Schematic representation of identifying drug targets by global thermal proteome profiling of 22Rv1 cells and 433‐3β (ligand) by liquid chromatography tandem mass spectrometry. a–d represent four different proteins; b represents lanosterol synthase (LSS) or CYP51A1 and is bound by drug (433‐3β), potentially inhibiting it, thus stabilized from heat denaturation.

The binding targets of the lead compound 433‐3β were determined by thermal proteome profiling (TPP) as described [48, 49, 50] and schematically represented in Fig. 1. Briefly, 22Rv1 cells was treated with 15 μm 443‐3β for 24 h. Treated protein aliquots were differentially denatured and precipitated by heating to six elevated temperatures evenly distributed from 43 °C to 60 °C followed by ultracentrifugation. The quantities of the remaining folded proteins at each temperature were measured and compared with those of controls, which were treated similarly but with solvent only, using label‐free quantification by liquid chromatography tandem mass spectrometry (Waters nano‐ACQUITY UPLC system analyzed on a coupled Thermo Scientific Orbitrap Fusion Lumos). Protein abundance ratios between the treated samples and the vehicle controls were measured by comparing the MS1 peak volumes of peptide ions, whose identities were confirmed by searching MS2 spectra against a UniProt human reference proteome using Sequest algorithm [51] and MS Amanda algorithm [52] described previously. VNPP443‐3β‐bound proteins were expected to show a different thermostability in comparison with the unbound proteins during the thermal denaturation. Binding target candidates showing closely related activities were selected for equilibrium unfolding modeling using Eqn (1) [53].

(1) $$f\left(T\right)=\frac{1-p}{1+e^{-\left(\frac{a}{T}-b\right)}}+p$$

Equation (1): Equilibrium unfolding equation. *T* is the temperature and *a*, *b*, and *p*, are constants derived from regression.

The most promising candidate binding targets that fit the equilibrium unfolding model and showed significant shift in melting point (Δ*T*_m_) were selected for molecular docking. Meanwhile, comparative chemoproteomics studies were performed as described previously [54] to compare the global proteome differential expressions induced by the 443‐3β treatment in PCa cells using perseus software (perseus v2.0.11; Max Planck Institute of Biochemistry, Martinsried, Germany) followed by Qiagen Ingenuity Pathway Analysis (IPA).

### 2.9 Statistical analysis

Statistical analysis was performed using a one‐way ANOVA and Student's *t*‐test using graphpad prism 9.0 software (GraphPad Software, Inc.). Probability values of *P < 0.05, **P < 0.01, ***P < 0.001, and ****P < 0.0001 were considered statistically significant. The values are presented as mean ± SEM of three or more independent experiments.

## 3 Results

### 3.1 Thermal proteome profiling and CETSA demonstrate VNPP433‐3β binds LSS and CYP51A1 *in vitro* and *in situ*

**Fig. 2:**
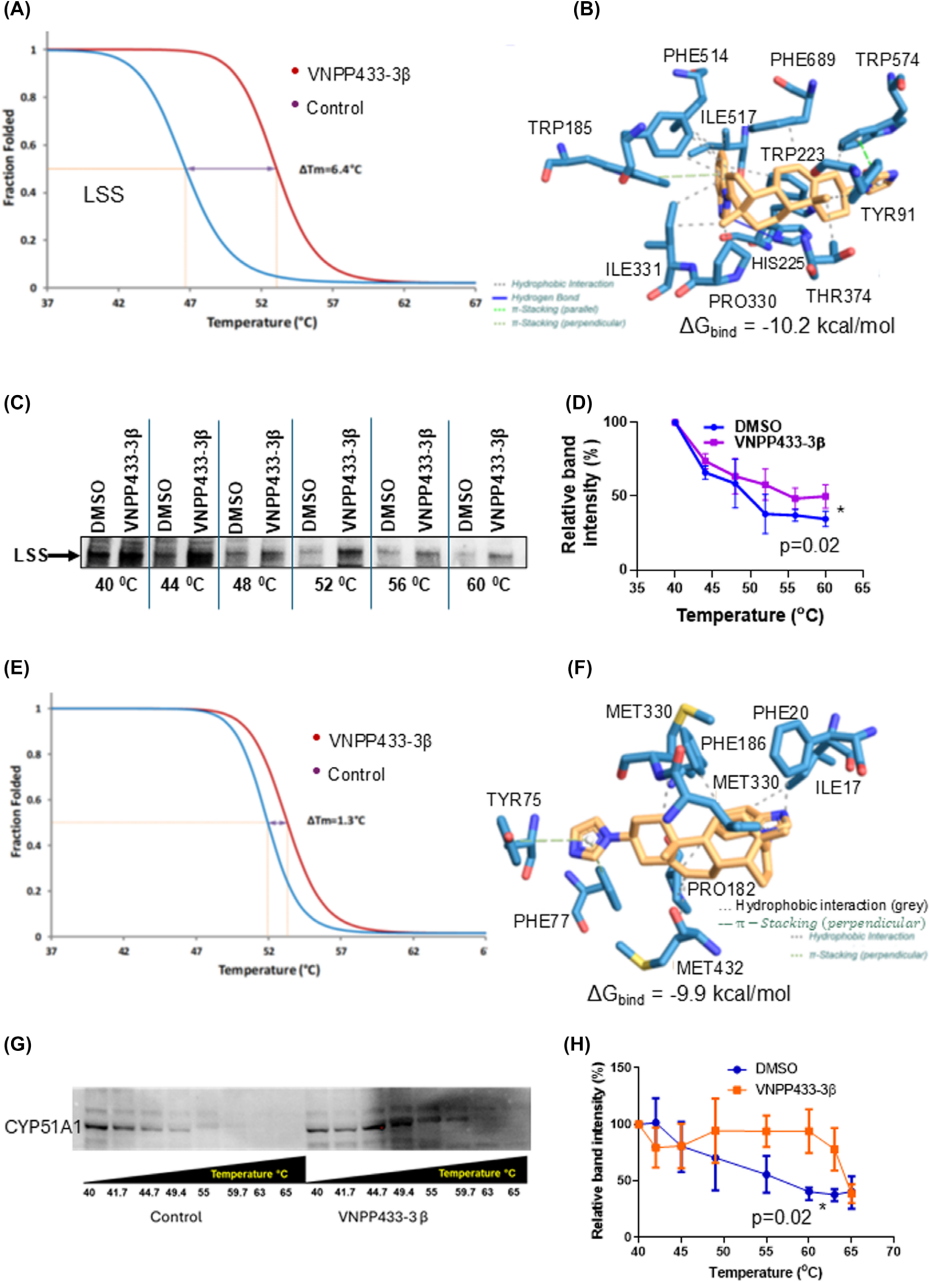
VNPP433‐3β binds lanosterol synthase (LSS) *in vitro* and *in situ*. (A) Thermal proteome profiling using high‐definition mass spectrometry (HDMS) reveals distinct melting curves for the LSS protein in the presence of 433‐3β. Lanosterol synthase exhibited a substantial shift in melting temperature (Δ*T*_m_) of 6.4 °C when 22Rv1 cells were treated with 433‐3β. (B) 433‐3β was docked into the active site of LSS using *in silico* molecular docking, showing a strong binding energy (Δ*G*) of −10.2 kcal·mol^−1^. Interacting amino acid residues in the binding pocket of LSS upon binding with 433‐3β *in silico* are shown. (C) Representative immunoblot showing thermal stabilization of LSS upon ligand binding that was measured by cellular thermal shift assay (CETSA) in 22Rv1 cells. Aliquots of 1 × 10^6^ 22Rv1 cells in 100 μL were subjected to varying temperature as shown in presence of 433‐3β, cells were lysed by freeze–thaw three times and centrifuged. The proteins in supernatant were separated by SDS/PAGE and immunoblotted using anti‐LSS (*n* = 3). (D) Apparent melting curve of LSS: graph was plotted as a function of temperature used in heat treatment as shown in C using three independent experiments. (E) The binding of 433‐3β to lanosterol 14‐alpha demethylase (LDM, CYP51A1) was demonstrated by thermal proteome profiling, resulting in a thermal shift (Δ*T*_m_) of 1.3 °C. (F) Molecular docking of 433‐3β to lanosterol 14‐alpha demethylase demonstrates a strong binding *in silico* with a free energy of binding (Δ*G*) −9.9 kcal·mol^−1^. (G) Representative immunoblot of cellular thermal shift assay (CETSA) of CYP51A1 showing enhanced protein stabilization upon treatment with 433‐3β at higher temperature compared with the DMSO control (*n* = 3). (H) Apparent melting curve of CYP51A1 as a function of temperature from three independent experiments. All error bars represent SEM. (D) and (H) were analyzed by Student's *t*‐test in graphpad prism 9.0, *P < 0.05.

Differential thermal profiling of total proteins from 22Rv1 PCa cells treated with 15 μm 433‐3β (24 h) demonstrated a significant shift in fraction of natively folded lanosterol synthase (LSS) and lanosterol 14‐alpha demethylase (CYP51A1) against a temperature gradient. Interestingly, these two proteins playing important roles in CHOL metabolism also showed significant binding *in silico*. Over 3000 proteins measured, 81 proteins were found to be stabilized by 433‐3β while 89 were destabilized at varying degrees. LSS was significantly stabilized and ranked 3 in the list with Δ*T*_m_ of 6.4 °C (Fig. 2A and Table S1). Though we consider LSS as the major target of 433‐3β in the CHOL biosynthesis pathway due to higher Δ*T*_m_ (6.4 °C) compared with that of CYP51A1 (Δ*T*_m_ = 1.4 °C), we validated both the LSS‐433‐3β and CYP51A1‐433‐3β binding further using CETSA *in situ*. Due to structural similarity of the substrates, the potential binding of 433‐3β to CYP51A1 could provide an additional advantage in inhibiting the pathway. LSS also called oxidosqualene cyclase (OSC) catalyzes the conversion of (S)‐2,3‐oxidosqualene to a protosterol cation and finally to lanosterol, which is a key four‐ringed intermediate in CHOL biosynthesis in human [55]. Due to the enzyme's important role in CHOL biosynthesis, interest has grown in LSS inhibitors as drugs to lower blood CHOL, treat atherosclerosis and inhibit tumor growth recently [14]. LSS is a highly likely binding target of 433‐3β as it showed significantly large shift in melting temperature (Δ*T*_m_ = −6.4 °C) compared with the control (Fig. 2A). Furthermore, the *in silico* analysis by molecular docking of 433‐3β to LSS yielded a strong *in silico* binding energy of −10.2 kcal·mol^−1^, which is very significant and implies strong binding (Fig. 2B). The mass spectrometry proteomics data have been deposited to the ProteomeXchange Consortium via the PRIDE partner repository [56] with the dataset identifier PXD060338.

To further our understanding, we next studied the extent of 433‐3β's binding to LSS in live 22Rv1 cells by CETSA (Fig. 2C). At 40 °C, the amount of protein detected in control and treated cells was nearly the same, but as the temperature was gradually increased, a concomitant decline in protein levels was noted by immunoblotting and densitometry. Interestingly, 50% of protein in control was denatured by heating and thus precipitated at 50 °C while the treated cells retained 50% of native LSS until the temperature is raised to 55 °C, suggesting a Δ*T*_m_ of 5 °C *in situ* (Fig. 2D).

In addition, another enzyme in the pathway CYP51A1 (lanosterol 14α‐demethylase) was also stabilized albeit to a lesser extent with a Δ*T*_m_ of 1.3 °C (Fig. 2E and Table S1), which is significant, especially in the light of *in silico* docking studies that suggested high‐affinity binding with a predicted Δ*G*_bind_ of −9.9 kcal·mol^−1^ (Fig. 2F). The second candidate target CYP51A1 showing response toward 443‐3β is also directly involved in lanosterol metabolism. It participates in the synthesis of CHOL by catalyzing the removal of the 14α‐methyl group from lanosterol and thus is responsible for the essential demethylation step in the biosynthesis of sterols, which is regarded as the initial checkpoint in the transformation of lanosterol to other sterols that are widely used within the cell [57]. This target has also been proposed as a promising target for anticancer chemotherapy [58]. This enzyme was identified to be a binding target of compound 443‐3β with a significant *T*_m_ shift of 1.3 °C in comparison with control (Fig. 2E) and demonstrated a strong *in silico* binding energy of −9.9 kcal·mol^−1^ in molecular docking studies (Fig. 2F). Furthermore, the CETSA using anti‐CYP51A1 demonstrated significant stabilization of the protein by 433‐3β (Fig. 2G). The apparent melting curve of CYP51A1 (Fig. 2H) shows a ${T_{m}}_{50}$ (temperature at which 50% protein is denatured/melted) of 56 °C in control whereas 433‐3β binding stabilized CYP51A1 from denaturing and increased the ${T_{m}}_{50}$ to 64 °C (Fig. 2H). The large Δ*T*_m_ of 8 °C observed is probably due to the inherent stable nature of the protein, which displayed a higher ${T_{m}}_{50}$ of 56 °C in control itself. The CETSA results further support the binding of 433‐3β to CYP51A1, consistent with the molecular docking studies and thermal proteome profiling by mass spectrometry.

### 3.2 VNPP433‐3β induces cellular proteomic changes in prostate carcinoma cells

**Fig. 3:**
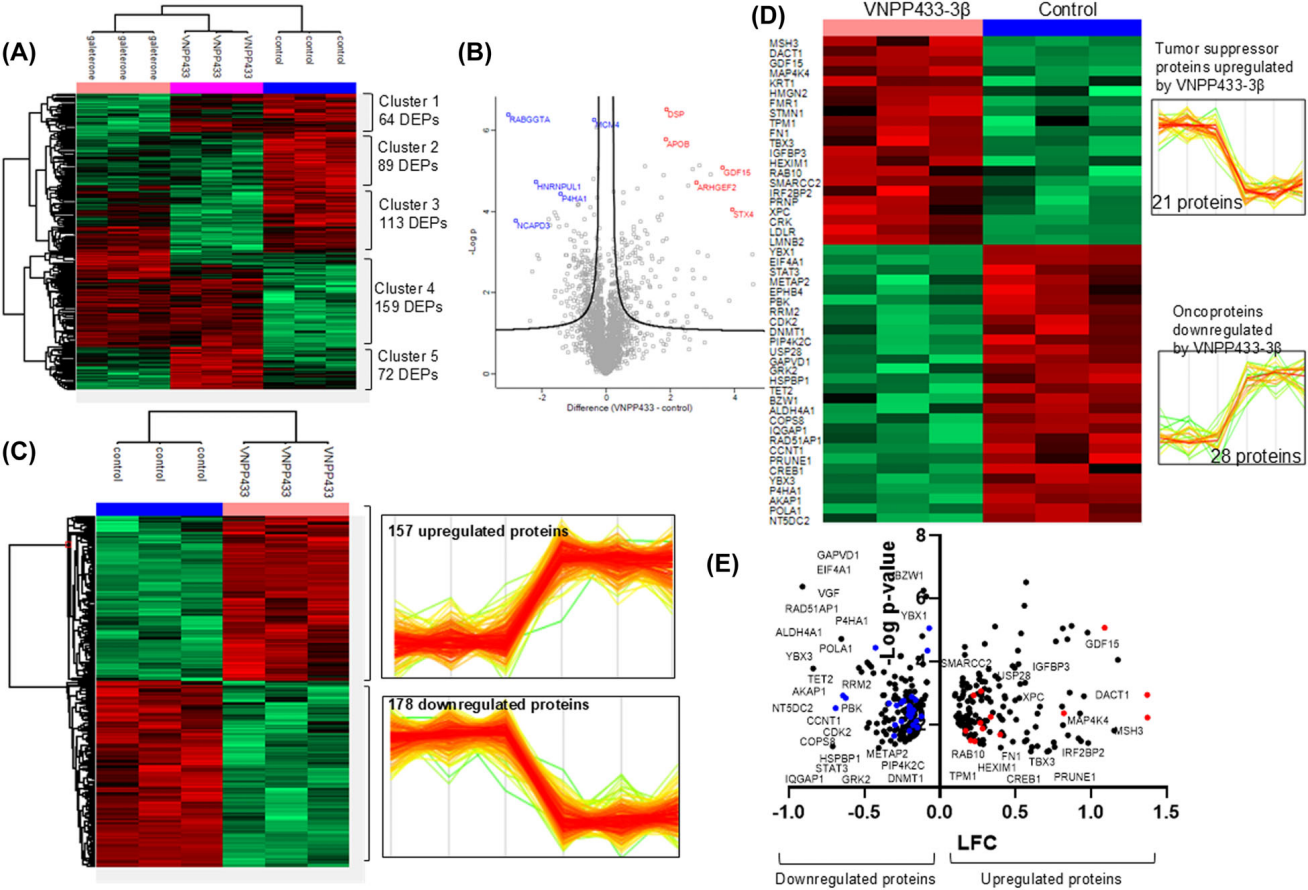
VNPP433‐3β induced significant proteomic changes in human prostate carcinoma epithelial cell line 22Rv1. (A) Unsupervised hierarchical clustering of differentially expressed proteins (DEPs) identified in 433‐3β and its parental compound galeterone (GAL) compared with control [ANOVA, false discovery rate (FDR) < 0.05]. (B) Volcano plot of DEPs identified in 433‐3β treatment compared with control. (C) Heat map showing differential expression of proteins upon 433‐3β treatment. A total of 157 proteins were upregulated while 178 were downregulated. (D, E) Many oncoproteins were downregulated by 433‐3β while several tumor suppressor proteins upregulated in PCa cells. Curated proteins relevant to cancer of prostate or other organs in humans. Heat map (D) and volcano plot (E) showing upregulation of 21 established or potential tumor suppressor proteins and downregulation of 28 oncoproteins upon treating PCa cells with 433‐3β. Red and blue dots in the volcano plot represent upregulated tumor suppressor proteins and downregulated oncoproteins, respectively.

Treating human prostate carcinoma epithelial cell line 22Rv1 cells with 433‐3β induced significant changes in global proteome when studied with high‐definition mass spectrometry (HDMS) (Table S2). We found that 433‐3β caused upregulation (twice or more) of 157 proteins while 178 proteins downregulated. Figure 3A,B along with Table S3A–C represent the heat map and volcano plot, respectively, of differential protein expression obtained by treating the cells with 433‐3β and its parent compound GAL compared with the untreated control cells and are grouped into five clusters. Figure 3C represents the differential expression of proteins in control versus 433‐3β and the trend of individual proteins' upregulation or downregulation. Interestingly, 28 major oncoproteins, including CDK2 implicated in PCa progression [59] and STAT3 critical for PCa cell proliferation and metastasis [60, 61], are downregulated by 433‐3β while 21 tumor suppressor proteins, especially TPM1, a known PCa suppressor [62] upregulated compared with the control (Fig. 3D,E).

### 3.3 Ingenuity pathway analyses of proteome demonstrate VNPP433‐3β modulates several oncogenic pathways in PCa cells

**Fig. 4:**
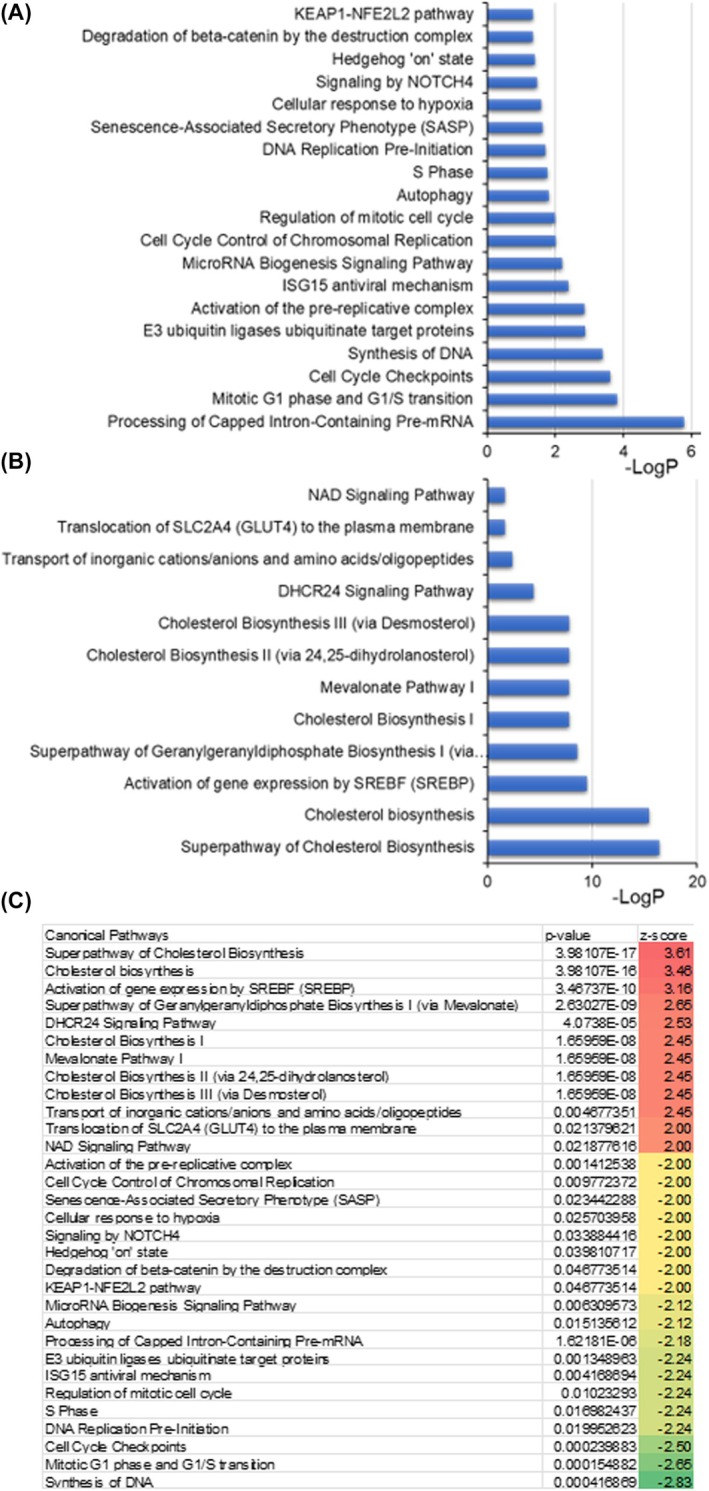
VNPP433‐3β significantly altered key canonical pathways in prostate cancer (PCa) cells. (A) Downregulated and (B) upregulated pathways in 22Rv1 cells treated with 433‐3β (*P* < 0.05, *z* > 2 or *z* < −2). DNA repair and replication in PCa cells are affected leading to arrest of G2/M transition of mitotic cell cycle. Notably, the mitochondrial electron transport is impaired and transcription by RNA polymerase II, the enzyme that synthesizes mRNA is decreased 15‐fold. Other aspects of cell cycle control, such as G1/S transition of mitotic cell cycle, are compromised. Note that some enzymes in cholesterol (CHOL) biosynthesis pathways were upregulated as cellular feedback inhibition response in an attempt to compensate and overcome the inhibition of the pathway, a strategy of cells' survival (see discussion). (C) The major pathways altered by 433‐3β along with its *Z*‐score and *P*‐value are summarized. A positive or negative *Z*‐score refers to upregulation or downregulation, respectively.

Concurrent with our previous studies [25, 35], global proteome analyses by ingenuity pathway analysis (IPA) shows that 433‐3β modulated several metabolic pathways and upstream regulators in PCa cells. Our proteome data suggest that 433‐3β exceedingly (32‐fold) downregulated the proteins involved in the ‘G1/S transition of mitotic cell cycle’, which is a crucial checkpoint in the cell cycle (Table S2). Likewise, hypoxia and hypoxia‐driven transcription favors tumor cells by modulating tumor microenvironment [63]. Interestingly, ‘transcription from RNA polymerase II promoter in response to hypoxia’ is significantly downregulated upon 433‐3β treatment. Notably, cell cycle progression was blocked at several steps, including mitotic G1 phase and G1/S transition, DNA replication preinitiation, synthesis of DNA, mitotic cell cycle, S‐phase, and regulation of mitotic cycle. Additionally, in cellular response to hypoxia, a key alternative pathway, the cancer cells invoke for survival is inhibited. Furthermore, Notch4 signaling, a key hallmark of cancer stem cells, is inhibited by 433‐3β (Fig. 4A). Androgen depletion therapy (ADT)‐sensitive prostate cancer cells stall at G0 of the cell cycle when deprived of androgen [64, 65, 66]. As 433‐3β is known to interrupt AR signaling by promoting the degradation of AR [25], it is expected that the various cell cycle processes, such as DNA synthesis/S‐phase, mitotic G1 phase and G1/S transition, that steer the cell cycle forward are inhibited as demonstrated by 433‐3β (Fig. 4A). Mechanistically, systematic progression from G1 to mitosis is largely controlled by different types of cyclin and corresponding cyclin‐dependent kinase (cdk) complexes (reviewed by Lee and Sicinski) [67]. Interestingly, AR signaling triggers mTOR‐dependent translation of cyclins D1 and D3 [68] but are suppressed in ADT‐responsive prostate cancer cells when deprived of steroids [66, 68]. AR signaling upregulates cyclins D1 and D3 leading to a spike in its levels, which further activates CDK4 and CDK6 that phosphorylate and inactivate retinoblastoma protein (Rb) to begin the S‐phase [68] but is inhibited by 433‐3β (Fig. 4A).

**Fig. 5:**
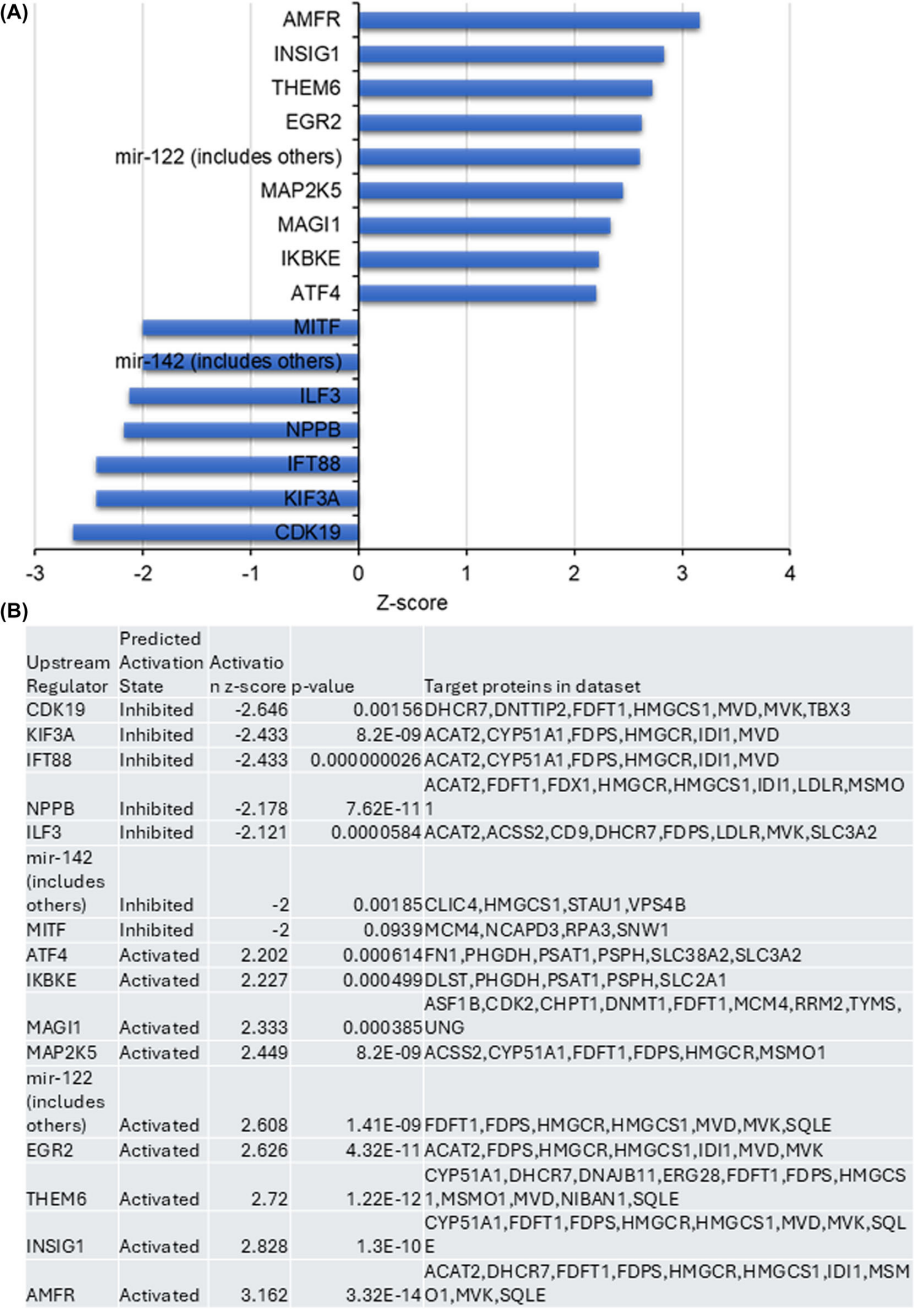
VNPP433‐3β altered the expression of key upstream regulators. (A) The upstream regulator proteins significantly altered by 433‐3β in prostate cancer (PCa) cells as inferred from high‐definition mass spectrometry (HD‐MS) proteomic studies (*P* < 0.05, *z* > 2 or *z* < −2). (B) Sixteen inferred upstream regulators along with its target genes and *Z*‐score as described in A. Note the elevated levels of AMFR and Insig1 both responsible for maintaining cholesterol (CHOL) homeostasis by lowering the expression of CHOL biosynthesis pathway genes.

Contrary to the decreasing effects on CHOL levels in PCa cells and tumor tissue, proteomic data show that some enzymes in the CHOL biosynthesis pathway are upregulated, resulting in the accumulation of those enzymes (Fig. 4B,C). This phenomenon has been documented in other studies, with reports indicating accumulation of CHOL biosynthesis pathway enzymes by as much as 200‐fold when a key pathway enzyme is inhibited [69, 70, 71]. However, autocrine motility factor receptor (AMFR or gp78) and insulin induced gene 1 (Insig1), two major upstream regulators of CHOL homeostasis responsible for lowering the levels of CHOL [72, 73], are significantly upregulated (Fig. 5A,B). AMFR plays a critical role in regulating CHOL biosynthesis by sterol‐dependent ubiquitination and proteasomal degradation of HMGCR (HMG CoA reductase enzyme), a key rate‐limiting enzyme in CHOL biosynthesis [74, 75]. Similarly, Insig1 is involved in the regulation of CHOL homeostasis by retaining sterol regulatory element‐binding proteins (SREBPs) in the endoplasmic reticulum (ER). Insig1 binds to SREBP cleavage‐activating protein (SCAP) if the CHOL level is high and prevents the activation of SREBPs. This in turn decreases the expression of CHOL biosynthesis genes, including HMG CoA reductase, a key regulatory enzyme in the pathway thereby preventing a spike in CHOL levels [75, 76].

Other key upstream regulators upregulated by 433‐3β treatment are EGR2 and Mag1, an apoptosis inducer and tumor suppressor, respectively [77, 78]. CDK19 that activates proliferation of hematopoietic [79] and potential cancer stem cells are downregulated by 433‐3β. Kif3A that promote PCa [12], NPPB, a potential oncogene [80], and ILF‐3, a cancer stem cell upstream regulator [81], are downregulated by 433‐3β treatment (Fig. 5A,B). These findings are in conjunction with our previous studies using next‐generation RNA‐sequencing and transcriptome analyses where we reported modulation of several pathways, including inhibition of PCa stem cells by 433‐3β [25, 35].

### 3.4 VNPP433‐3 decreases CHOL levels in PCa cells *in vitro* and *in vivo*

**Fig. 6:**
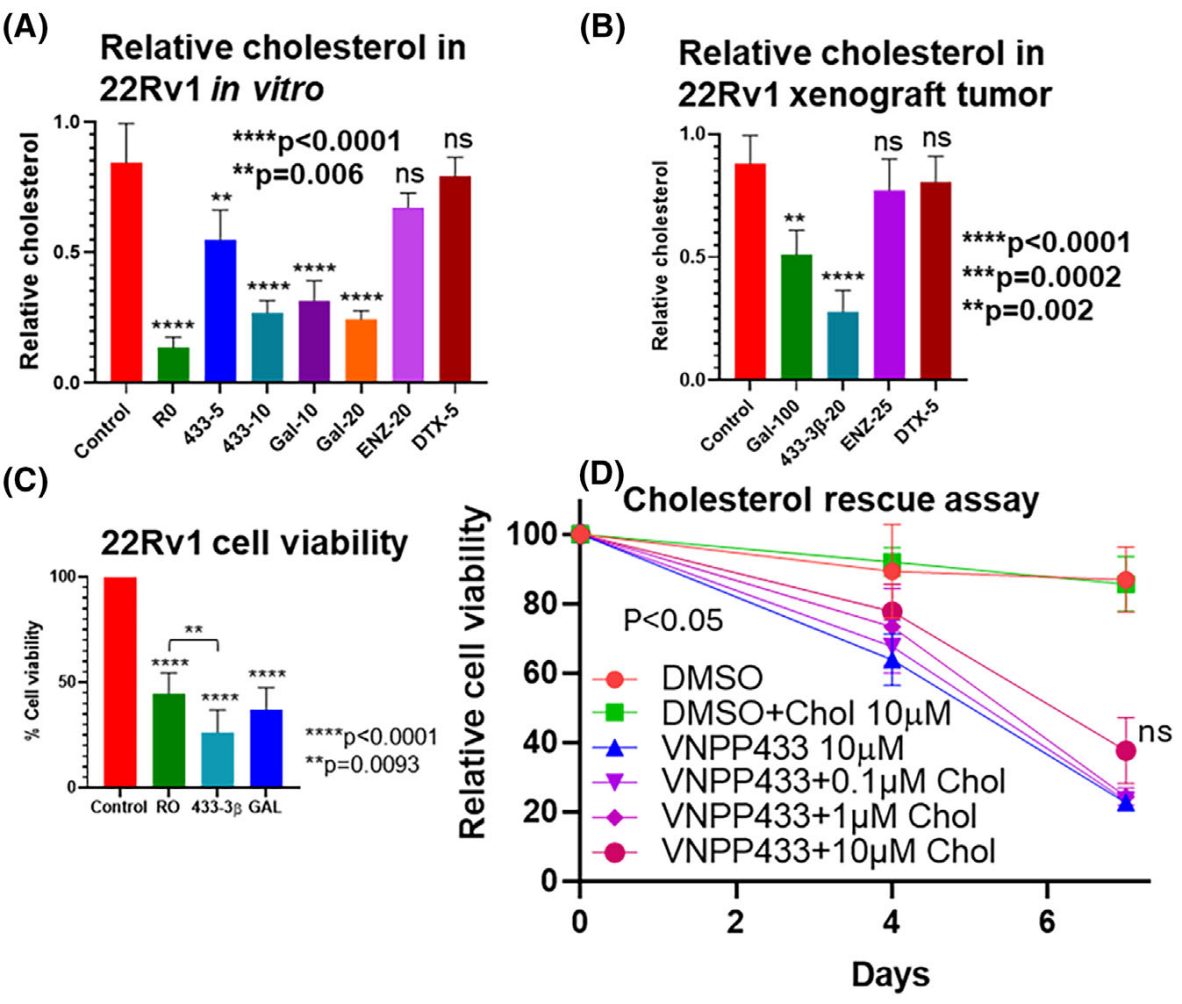
VNPP433‐3β lowers cholesterol (CHOL) levels in prostate cancer cells *in vitro* and xenograft tumor *in vivo*. Relative total CHOL (free + esteryl) measured using Amplex™ red CHOL assay in 22Rv1 cells treated with various treatments *in vitro* (A) and *in vivo* xenograft tumor tissue (B). RO (RO48‐8071)—1 μm; 433‐3β‐5, 10 μm *in vitro* and 20 mg·kg^−1^ *in vivo*; galeterone (GAL)—10, 20 μm *in vitro* and 100 mg·kg^−1^ body weight *in vivo*; enzalutamide (ENZ)—20 μm and 25 mg·kg^−1^ body weight *in vivo*; docetaxel (DTX)—5 μm *in vitro* and 5 mg·kg^−1^ *in vivo*. (C) MTT cell viability assay following treatment with lanosterol synthase (LSS) inhibitor RO (1 μm). Low levels of CHOL observed in galeterone‐ (10 μm) or 433‐3β‐treated (10 μm) cells is not due to androgen receptor (AR) inhibition as ENZ, a known AR antagonist did not yield significant reduction of CHOL *in vitro* and *in vivo*. (D) CHOL rescue assay: supplementing PCa cells with externally added CHOL did not significantly rescue the cells from GAL or 433‐3β, indicating other mechanisms of action beyond CHOL‐lowering effect. The experiment was performed twice independently with five technical replicates in each experiment. All error bars represent SEM. The groups are statistically analyzed by one‐way ANOVA in graphpad prism 9.0. *P* < 0.05, ns—not significant, ****P < 0.0001, ***P < 0.001, **P < 0.01, *P < 0.05.

Following treating the PCa cells *in vitro* and oral administration of 433‐3β to mice bearing 22Rv1 xenograft tumor, the total CHOL level in the cells and tumor tissue was measured by Amplex™ red CHOL assay. The CHOL level was significantly lowered in the cultured PCa cells when treated with 5 or 10 μm 433‐3β, 10 or 20 μm GAL and known CHOL biosynthesis inhibitor RO (RO0488971, 1 μm) (Fig. 6A). A similar finding was observed in CHOL levels of xenograft tumor tissue where GAL‐ and 433‐3β‐treated animals showed decreased levels of CHOL in tumor (Fig. 6B) with concomitant reduction in tumor volume [38]. Low levels of CHOL observed in 433‐3β‐treated cells is not due to AR inhibition as ENZ, a known AR antagonist did not yield significant reduction of CHOL *in vitro* and *in vivo*. Furthermore, we compared the cell viability after treating PCa cells with 433‐3β, GAL or RO, all of which inhibited PCa cell growth at varying degree *viz* 73.7%, 62.9%, and 55.3%, respectively (Fig. 6C). Next, we supplemented the growth medium with CHOL to determine whether it could rescue the PCa cells from cell death. We did not observe significant decrease in cell death at 10 μm CHOL, suggesting that other cytotoxic mechanisms are at play when treated with GAL or 433‐3β (Fig. 6D).

## 4 Discussion

The role of CHOL homeostasis in PCa, whether active or passive, has been a topic of interest for decades. However, significant breakthroughs have emerged recently with the advent of high‐throughput transcriptome and proteomic research methods, combined with the availability of various genetic and *in vivo* models. Growing evidence from transcriptomic, biochemical, *in vivo* and patients' clinical data suggests a strong correlation between elevated CHOL levels, both circulating and *intra tumorem*, and the incidence and severity of PCa [5, 6, 17, 82]. Previously we have demonstrated the anti PCa efficacy of 433‐3β in different models of PCa that represent CRPC and hormone‐dependent PCa [25]. As proof of concept of CHOL biosynthesis pathway interruption by 433‐3β, the *in vitro* and *in vivo* experiments are carried out using 22Rv1 cell line that represents the ‘difficult to treat’ metastatic CRPC as cholesteryl ester accumulation is correlated to high‐grade and metastatic PCa [83]. Additionally, we reported the major mechanism of action of 433‐3β that promotes proteasomal degradation of AR and Mnk1/2 in PCa cells, thus regulating AR‐driven oncogenic transcription and mRNA 5′ cap‐dependent deregulated protein synthesis mediated by eIF4E‐Mnk1/2 [25, 34]. Transcriptome analysis of PCa cells treated with 433‐3β showed modulation of several metabolic and signaling pathways leading to inhibition of tumor *in vivo* and cancer stem cells [25, 35]. Transcriptomics tends to be more routine than proteomics due to relative ease, cost, and reproducibility though the proteome best reflects cells' state and behavior [84].

Thermal proteome profiling data in this study showed that 433‐3β significantly binds and stabilizes lanosterol synthase (LSS), a rate‐limiting enzyme in CHOL biosynthesis and lanosterol 14α‐demethylase (LDM or CYP51A1), another key enzyme in sterol and CHOL biosynthesis pathway. Binding of 433‐3β to these enzymes is an interesting discovery as GAL was originally developed as a CYP17 inhibitor, another enzyme in biosynthesis of steroid precursors of CHOL biosynthesis [85, 86], and was subsequently identified as an AR‐downregulating agent [87]. Later, its precise mechanism was characterized, revealing that it stimulates proteasomal degradation of AR thereby decreasing its level in PCa cells [88]. TPP data were further validated *in silico* and *in situ* by molecular docking and CETSA that revealed strong free energy of binding and large shift in apparent melting temperature, respectively. Consequently, GAL and 433‐3β significantly decreased the total CHOL levels in PCa cells and tumor tissues of treated mice. The CHOL rescue assay further reiterates additional mechanisms of action beyond the CHOL‐lowering effect of 433‐3β. It is relevant to state here that a recent study reported the interaction of CYP17A1 inhibitors, abiraterone and GAL with human sterol 14α‐demethylase (CYP51A1) [89].

The accumulation of some enzymes in CHOL biosynthesis pathway upon treating PCa cells with 433‐3β observed in comparative proteomics study is not unusual. Contradictory to the decreased CHOL levels in treated cells and tumor tissue, proteomic data reveal that some enzymes in the CHOL biosynthesis pathway are upregulated, leading to their accumulation (Fig. 4B,C). This phenomenon has been documented in other studies, with reports indicating an upregulation of CHOL biosynthesis pathway enzymes by as much as 200‐fold when one of the pathway enzymes is inhibited [69, 70, 71]. As the enzyme levels are controlled by gene expression and protein degradation mechanisms, accumulation of enzymes in a pathway when it is blocked is not typically a direct consequence of the blockage itself. However, there are some specific instances where enzyme levels may increase due to increased transcription and translation of genes encoding enzymes in the pathway in response to pathway inhibition, primarily due to regulatory feedback mechanisms in cells' attempt to compensate and overcome the inhibition. Additionally, some drugs, especially that of steroid class, can induce CYP enzymes (drug‐induced enzyme induction) that are also part of CHOL biosynthesis [90, 91]. However, besides limiting precursor for androgen synthesis, achieving a reduced CHOL level is beneficial for a better treatment outcome in PCa as demonstrated by several studies [6, 17].

In summary, our TPP and biochemical data show that 433‐3β lowers CHOL in PCa *in vitro* and *in vivo*. Using comparative proteomics, we demonstrate that 433‐3β modulated the expression of several upstream regulator proteins thereby exerting its ancillary effects in inhibiting PCa and PCa stem cells as demonstrated by diminished Notch4 signaling, ILF3 and CDK19 required for stemness and potentially PCa recurrence as we previously reported using transcriptome analysis [25, 35]. In concordance with our previously reported transcriptome data, the comparative proteomics reiterate that several sub‐pathways contributing to cell cycle progression, such as S‐phase, DNA synthesis, mitotic G phase, and G1‐S transition, are inhibited by 433‐3β in PCa cells.

## 5 Conclusion

The inhibition of CHOL biosynthesis serves as a significant ancillary effect of VNPP433‐3β, reinforcing its overall inhibition of PCa and expected to deliver a multipronged effect in inhibiting PCa cells as a single agent and further reiterates its potential for treating patients with all stages of PCa.

## Conflict of interest

Vincent C. O. Njar is the lead inventor of VNPP433‐3β, and the patents and technologies thereof are owned by the University of Maryland, Baltimore. Puranik Purushottamachar is the co‐inventor of VNPP433‐3β. No potential conflicts of interest were disclosed by the other authors.

## Author contributions

ET, RST, and VCON conceptualized and designed the study. ET, RST, WH, and PP performed the experiments and acquired data. ET, RST, PP, WH, MMW, MAK, YZ, NA, B‐DW, DW and VCON analyzed the data. ET, RST, and VCON wrote the manuscript. VCON supervised the entire study. All authors read and approved the final manuscript.

## Supporting information


**Table S1.** Relative abundance of proteins by thermal proteome profiling.
**Table S2.** Proteins in various biological pathways that are modulated by 433‐3β.
**Table S3.** Differentially expressed proteins identified by mass spectrometry upon treatment with 433‐3β.

## Data Availability

All datasets used and analyzed during the current study are presented here. The mass spectrometry proteomics data have been deposited to the ProteomeXchange Consortium via the PRIDE [56] partner repository with the dataset identifier/accession number PXD060338.
