# Supplementary material for: Thermal proteome profiling and proteome analysis using high‐definition mass spectrometry demonstrate modulation of cholesterol biosynthesis by next‐generation galeterone analog VNPP433‐3β in castration‐resistant prostate cancer
Source: Mol Oncol. 2025 Feb 26;19(8):2292–309. doi: 10.1002/1878-0261.70009 (PMC12330945; doi:10.1002/1878-0261.70009)
Supplement: Supplementary file 1 — Table S1. Relative abundance of proteins by thermal proteome profiling. Table S2. Proteins in various biological pathways that are modulated by 433‐3β. Table S3. Differentially expressed proteins identified by mass spectrometry upon treatment with 433‐3β. [file MOL2-19-2292-s001.zip › TablesS1-S3_Legends.docx]

**Table S 1.** **Relative abundance of proteins by thermal proteome profiling.** Over 3000 proteins studied in 22RV1 cells by thermal proteome profiling, top 200 are ranked for potential stabilization or destabilization by 433-3β. Note that LSS is ranked 3 and CYP51A1 ranked 117 in the list.

Key to read columns: Columns A- UniProt accession number of the protein; column B- Name of the protein and species-specific description (OS- Homo sapiens; OX- 9606" - the unique numerical code assigned to "Homo sapiens" in the NCBI database.; GN- the specific name of the gene; PE (Protein Existence), a score of 1 denotes ‘high confidence’; SV (sequence version)- the version number of the gene sequence to show potential updates to the sequence data. Column C- software-generated simulation of potential stabilization/ destabilization of the protein, which is not informative in this table. Columns D-I: refers to the relative abundance of protein (control vs VNPP433-3β) detected at different temperature treatment as mentioned. Columns J-N: stabilization/destabilization score at each temperature range as mentioned in first row. A negative value represents destabilization (green) and a positive values demonstrates stabilization of the protein upon ligand binding. O- overall stabilization score; P- stabilization score; Q- destabilization score; R-S- main and effective scores; T- validation of numeric score based on P and Q; V-CC- Columns represent relative abundance of a protein at a given condition as specified in the first row.

**Table S2.** **Proteins in various biological pathways are modulated by 433-3β.** Biological processes of proteins decreased by 433-3β as demonstrated by proteomics study. Note that many biological processes such as G1/S transition of mitotic cell cycle, Transcription in G1/S transition of mitotic cell cycle, DNA replication and repair responsible for cell cycle progression are inhibited at varying levels.

**Supplementary Table 3. Differentially expressed proteins identified by mass spectrometry upon treatment with 433-3β**. Table S3-A: All proteins identified from the proteomics data. The first row of each column provides the description.

Table S3-B: Differentially expressed proteins identified by ANOVA (FDR < 0.05).

Table S3-C: Differentially expressed proteins identified by t-test (FDR < 0.05) in 433-3β-treated 22RV1 cells.
